# PALLIATIVE CARE... WHAT’S THAT? : MEDICAID PATIENT-IDENTIFIED BARRIERS TO PALLIATIVE CARE

**DOI:** 10.1093/geroni/igz038.3342

**Published:** 2019-11-08

**Authors:** Deborah Hoe, Yu-Hsuan Wang, Kate Meyers, Susan Enguidanos

**Affiliations:** 1 University of Southern California, Los Angeles, California, United States; 2 Leonard Davis School of Gerontology, University of Southern California, Los Angeles, California, United States; 3 California Health Care Foundation, Oakland, California, United States

## Abstract

Multiple studies demonstrate most consumers do not know about palliative care. And, since January 2018, California’s Medi-Cal Managed Care patients have been eligible for palliative care services under Senate Bill 1004 (SB 1004). Yet, the uptake of palliative care services has been underwhelming. The purpose of this study is to explore patient-centered barriers to palliative care. We recruited 27 adult Medicaid patients suffering from advanced cancer, chronic obstructive pulmonary disease, congestive heart failure, or liver disease, from community-based sites in Los Angeles, and conducted semi-structured qualitative interviews. Each participant was asked questions to elicit their knowledge about, and perspectives on, palliative care as well as their preferred communication approaches for receiving a referral to palliative care. The interviews were audio-recorded and transcribed verbatim. We used a grounded theory approach to guide our analysis of primary themes. Our findings indicated that the barriers to palliative care referrals among this population included lack of knowledge about palliative care and available services; the reliance on, and trust in, primary care physicians for information; language and cultural barriers; and patient believing they are neither old enough nor sick enough to need palliative care. This population also preferred direct contact, with in-person consultations more favorable than telephone calls. These findings emphasize the critical role primary care physicians play in advocating for safety net patients and the necessity for culturally sensitive education about palliative care. Promoting knowledge and understanding of palliative care among both primary care physicians and consumers is vital to ensuring access to care.

